# Irisin: A Potentially Fresh Insight into the Molecular Mechanisms Underlying Vascular Aging

**DOI:** 10.14336/AD.2023.1112

**Published:** 2023-11-20

**Authors:** Yinghui Wang, Manying Wang, Yuehui Wang

**Affiliations:** ^1^Department of Geriatrics, Jilin Geriatrics Clinical Research Center, The First Hospital of Jilin University, Changchun, Jilin, China.; ^2^Research Center of Traditional Chinese Medicine, The Affiliated Hospital to Changchun University of Chinese Medicine, Changchun, China.

**Keywords:** vascular aging, irisin, senescence, AMPK

## Abstract

Aging is a natural process that affects all living organisms, including humans. Aging is a complex process that involves the gradual deterioration of various biological processes and systems, including the cardiovascular system. Vascular aging refers to age-related changes in blood vessels. These changes can increase the risk of developing cardiovascular diseases, such as hypertension, atherosclerosis, and stroke. Recently, an exercise-induced muscle factor, irisin, was found to directly improve metabolism and regulate the balance of glucolipid metabolism, thereby counteracting obesity and insulin resistance. Based on a growing body of evidence, irisin modulates vascular aging. Adenosine monophosphate-activated protein kinase (AMPK) serves as a pivotal cellular energy sensor and metabolic modulator, acting as a central signaling cascade to coordinate various cellular processes necessary for maintaining vascular homeostasis. The vascular regulatory effects of irisin are closely intertwined with its interaction with the AMPK pathway. In conclusion, understanding the molecular processes used by irisin to regulate changes in vascular diseases caused by aging may inspire the development of techniques that promote healthy vascular aging. This review sought to describe the impact of irisin on the molecular mechanisms of vascular aging, including inflammation, oxidative stress, and epigenetics, from the perspective of endothelial cell function and vascular macroregulation, and summarize the multiple signaling pathways used by irisin to regulate vascular aging.

## Introduction

**Figure 1.**
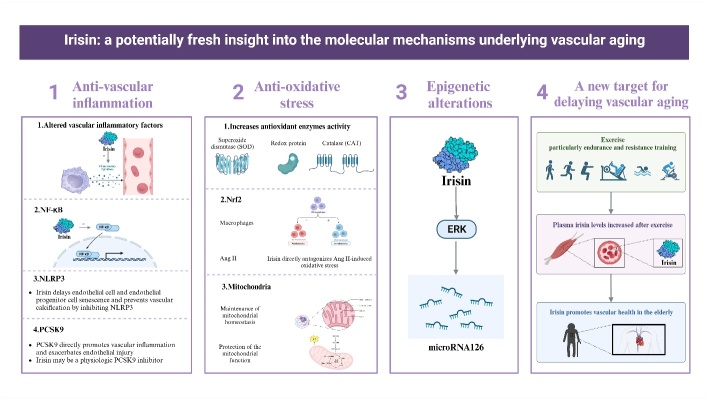
**Outline of irisin regulation of vascular aging**. Created with BioRender.com

Aging is a natural biological process in which the body undergoes physiological and morphological changes. In general, aging indicates a loss of defense, reactivity, and adaptability, which increases the risk of disease and eventually, mortality. Studies performed in recent decades have provided compelling evidence that exercise contributes to the regenerative features of enhanced tissue growth and regeneration and avoids degenerative diseases. Therefore, exercise is currently acknowledged as a crucial non-pharmacological strategy for the prevention, treatment, and rehabilitation of cardiovascular diseases (CVDs). Irisin, a myokine expressed in the skeletal muscle during exercise, leads to the favorable benefits of exercise at the molecular level, such as the maintenance of cardiovascular youth. In this review, we will discuss the beneficial effects of irisin on vascular aging through inflammation, oxidative stress, and epigenetics (Fig. 1).

### Irisin

**Figure 2.**
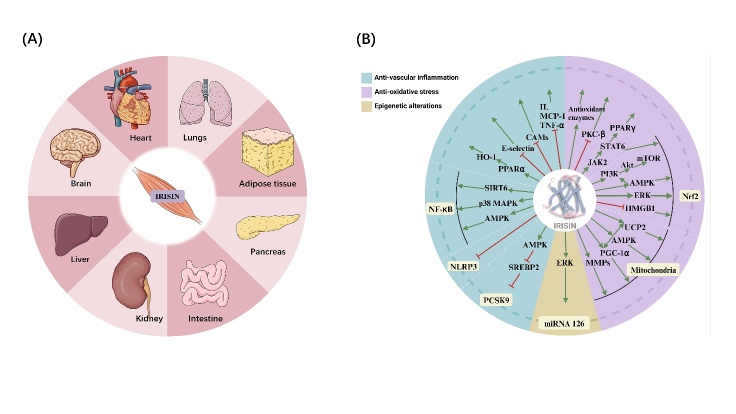
**Irisin plays a role in numerous organs and exhibits a combined vasoprotective effect through several routes. (A)** The heart, brain, liver, kidney, intestine, pancreas, adipose tissue, and lungs are affected by irisin, which is secreted by the muscle. **(B)** Irisin is involved in multiple signaling pathways that regulate vascular aging. Abbreviations: IL- interleukin; MCP-1- macrophage chemoattractant protein-1; TNF-α- tumor necrosis factor-α; CAMs- cell adhesion molecules; SIRT6- Sirtuin 6; MAPK- mitogen-activated protein kinase; AMPK - Adenosine monophosphate-activated protein kinase (AMPK); ERK- extracellular regulated protein kinases; MMPs- matrix metalloproteinases; PGC-1α- Peroxisome proliferator-activated receptor-γ coactivator-1α; UCP2- uncoupling protein 2; Akt- protein kinase B; mTOR- mammalian target of rapamycin; HMGB1- high-mobility group box 1; eNOS- endothelial nitric oxide synthase; PKC-β- protein kinase C-β; PCSK9- pro-protein convertase subtilisin/kexin type 9; SREBP2- sterolregulatory element binding protein 2; PPARγ- peroxisome proliferator-activated receptor gamma; JAK2- Janus kinase 2; STAT6-transduction and activator of transcription 6; HO-1- heme oxygenase-1.

In a case study performed in 2012 on peroxisome proliferator-activated receptor-γ coactivator-1α (PGC-1α) in muscle cells, PGC-1α was discovered, by chance, to induce muscle cells to secrete a glycosylated type I membrane protein called fibronectin type III domain-containing 5 (FNDC5), which is sheared into a soluble hormone, irisin, in the bloodstream [1, 2]. The skeletal muscle releases irisin, a glycosylated protein hormone composed of 112 amino acids, in response to shivering during exercise and cold exposure [1]. PGC-1α activates mitochondrial energy regulation, the oxidative energy supply of glucose and fat, and fat browning to modulate exercise-related responses by increasing FNDC5 expression and irisin release. Irisin is distributed in various tissues of the body and is involved in biochemical disorders, aging, inflammation, and neurogenesis [3, 4] (Fig. 2A). To date, most studies on FNDC5/irisin function have been conducted using mouse models or human cells. Most irisin concentrations are currently detected clinically using antibody-based methods (Western Blot, ELISA, Protein Liquid Chip Assay) and label-free methods (Quantitative Mass Spectrometry) [5]. Recombinant irisin administration or gene gain/loss of function has been used in preclinical investigations to modify irisin levels in animal and tissue engineering models [6-8].

The cardiovascular protective effects of exercise may be mediated by irisin. Exercise increases circulating irisin levels, and exogenous irisin treatment may mimic some of the benefits of exercise, including elevated energy expenditure and enhanced insulin sensitivity [2]. According to clinical studies, elevated irisin levels in Asian males are linked to a lower coronary atherosclerotic burden, and plasma irisin levels indicate the development of CVD [9]. Plasma iris levels are considerably lower in patients that suffer a heart attack, and a correlation exists between decreased iris levels and an increased risk of CVD [10]. Furthermore, numerous in vivo and in vitro studies revealed that exogenous irisin intervention alleviates endothelial damage, endocardial formation, and inflammatory oxidative stress, suggesting that it may slow the aging process of the vascular system [3] (Fig. 2B).

### Vascular Aging

A significant risk factor and driver of CVD in the elderly population is vascular aging, an age-related change in the vascular structure and function [11]. Vascular aging has two key characteristics: (1) arterial stiffness and (2) generalized endothelial cell (EC) dysfunction. With aging, arterial elastin degrades and stiffer collagen fibers accumulate, resulting in atherosclerosis [12]. Vascular calcification is a specific change; however, the precise process is unknown [13]. The clinical manifestations of increased oxidative stress and inflammatory cytokines, which are molecular biological processes for generalized endothelial dysfunction, include reduced antithrombotic capacity and vasodilatory function, resulting in atherosclerosis and thrombosis.

Chronic low-grade systemic and local inflammation, epigenetic changes, mitochondrial dysfunction, genomic instability, cellular senescence, loss of proteostasis, nutritional deficiencies, and stem cell failure are factors that affect aging of the vascular system [14]. The molecular biology of vascular aging comprises multiple, multifaceted processes of vascular aging. Inflammation and oxidative stress are among the most important mechanisms involved. Age-related endothelial failure is markedly caused by decreased bioavailability of nitric oxide (NO), a key mediator of vascular function preservation and anti-atherosclerosis [15]. NO is generated by l-arginine, catalyzed by endothelial nitric oxide synthase (eNOS), in the presence of the cofactor, tetrahydrobiopterin (BH4). According to recent studies, age-related increases in eNOS uncoupling and arginase (an enzyme that competes with eNOS for the l-arginine) activity are accompanied by a decline in BH4 availability, which inhibits NO release and causes the overproduction of the highly reactive superoxide anion (O_2_^-^) [16, 17]. Additionally, nicotinamide adenine dinucleotide phosphate (NADPH) oxidase activity increases with chronic inflammation and the high ROS concentration associated with aging, which elevates O_2_^-^and peroxynitrite (ONOO^-^) content [18]. A consequence of this age-related increase in ROS is the stimulation of vascular inflammation, activation of nuclear factor-kappaB (NF-κB), increased expression of pro-inflammatory factors, and vascular damage.

The objective of this review was to provide a comprehensive overview of vascular changes related to aging and the relationship between irisin and the processes that lead to vascular aging, such as chronic inflammation, oxidative stress, epigenetic changes, and mitochondrial protection. New insights into therapeutic targets for the prevention and treatment of vascular aging may be gained with more knowledge on the role of irisin.

## Anti-vascular inflammation

Age-related activation of the inflammatory process is a key pathological cause of macrovascular and microvascular aging, promoting atherosclerosis (AS) and microvascular dysfunction in various vascular disorders. Age-related vascular inflammation may be caused by various processes: (1) Multiple pro-inflammatory signals, such as NF-κB, are activated by oxidative stress in aging vessels [19]. These signals boost the production and release of several inflammatory cytokines and chemokines, and induce microvascular endothelial activation, leukocyte adhesion, and extravasation [14]. Increased levels of inflammatory substances stimulate oxidative stress in vascular cells. (2) Increased levels of pro-inflammatory cytokines and LDL-C trigger endothelial stimulation, resulting in a violent immunometabolic process of atherogenesis, which activates innate immune system effectors, including Toll-like receptors (TLRs) and the NOD-like receptor thermal protein domain associated protein 3 (NLRP3) inflammasome complex. Several studies have shown that NLRP3 causes an age-related systemic micro-inflammatory state [20].

Recent studies have shown that irisin reduces vascular aging by decreasing inflammation. Vascular aging may be caused by decreased irisin levels and vascular endothelial dysfunction working together in a causal manner [9, 21]. In addition, plasma/circulating irisin levels can be used to determine the prognosis of individuals with coronary artery disease and aid the evaluation of the severity of their coronary arteries [22]. In dyslipidemic rodents, daily injections of irisin (0.5 g) for 4 weeks improved AS lesions and inflammatory infiltration of the aortic surface, and decreased endothelial damage and vascular inflammation [23]. Similarly, EC apoptosis and endothelial injury were substantially decreased in diabetic mice administered irisin for 12 weeks (2 μg, twice weekly) [24].

Irisin inhibits the levels of cell adhesion molecules (CAMs), such as intercellular adhesion molecule 1 (ICAM-1), vascular cell adhesion protein 1 (VCAM-1), and E-selectin, which are elevated by dyslipidemia and reduces leukocyte and monocyte adhesion to the arterial wall surface [25]. Irisin also reduces the levels of vascular inflammatory factors. Endogenous irisin levels are lower in obese patients with diabetes, and a negative correlation exists between irisin and the pro-inflammatory cytokine, interleukin-6 (IL-6) [26]. Exogenous irisin treatment in atherosclerotic rodents substantially decreased plasma protein levels and the mRNA expression of IL-6, macrophage chemoattractant protein-1 (MCP-1), ICAM-1, and VCAM-1 [23]. Irisin was also found to reduce vascular pro-inflammatory factor levels in human umbilical vein endothelial cells (HUVECs) treated with oxidized low-density lipoprotein (ox-LDL), including tumor necrosis factor-α (TNF-α), MCP-1, interleukin-1α, IL-6, and interleukin-18 [23, 27].

**Figure 3.**
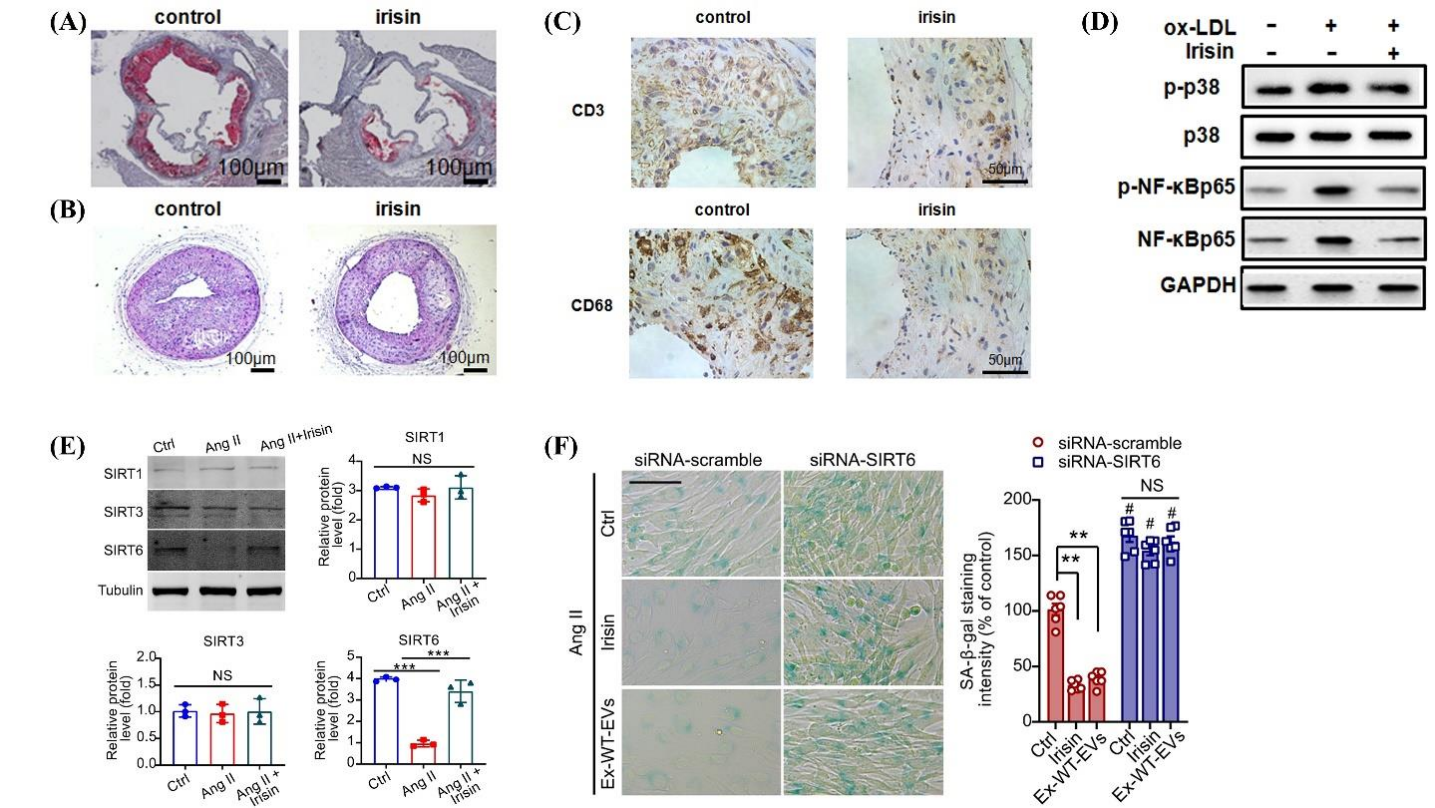
**Anti-inflammatory effect of irisin on blood vessels- NF-κB. (A)** Atherosclerotic lesions in the aorta of Apo E-deficient mice after 8 weeks of irisin treatment were identified using Oil-Red O staining. **(B)** Apo E-deficient mice were treated with or without irisin for 28 days after a partial carotid artery ligation. Hematoxylin-eosin staining was used to identify cross-sections of carotid arteries. **(C)** The effect of irisin on inflammatory infiltration in partially ligated carotid arteries, and images of CD3 and CD68 immunohistochemical staining in partially ligated carotid arteries. **(D)** Effect of irisin on ox-LDL-induced inflammation in HUVECs. The expression levels of p-p38 MAPK, p-NF-κB p65, and NF-κB p65 were analyzed via protein blotting. **(E)** Irisin treatment increases SIRT6 protein levels in cultured vascular smooth muscle cells but does not affect SIRT1 or SIRT3 protein levels. **(F)** Impact of exercise-induced extracellular vesicles or recombinant irisin on AngII-induced vascular smooth muscle cell senescence in wild-type mice. Abbreviations: Apo E-apolipoprotein E; HUVECs- human umbilical vein endothelial cells; AngII- Angiotensin II. (A-D) Reproduced with the permission of Ref.[23], Copyright of C 2016, PLoS One. (E-F) Reproduced with the permission of Ref.[28], Copyright of C 2022, European Heart Journal.

### NF-κB

NF-κB is an excellent indirect target for irisin because it is an important pro-inflammatory factor in the vascular aging process (Fig. 3). According to recent studies, FDNC5/irisin-rich extracellular vesicles (EVs) exhibit potent anti-vascular sclerosis and anti-aging effects in vivo and in vitro by upregulating FNDC5/irisin in DnaJb3/heat shock proteins 40 (Hsp40) chaperone-dependent sirtuin 6 (SIRT6) protein expression [28]. SIRT6 is a chromatin-associated deacetylase in the nucleus that inhibits the control of insulin-like growth factor (IGF)-protein kinase B (Akt) signaling and deacetylation of histone H3 at lysine 9 (H3K9), and exhibits cardioprotective effects [29, 30]. SIRT6 also prevents inflammatory and degenerative illnesses by blocking the transcription of NF-κB-dependent receptors [29]. Thus, by upregulating SIRT6 to prevent the aging of VSMCs and preserve vasodilatory capacity [31], irisin acts as an anti-aging agent in the vascular system [32].

By reducing ROS/p38 mitogen-activated protein kinase (MAPK)/NF-κB signaling, irisin reduces endothelial injury and inflammation, and prevents foam cell development and monocyte adhesion caused by ox-LDL [33]. Exogenous irisin delivery decreases mitogen-activated protein kinase (MAPK) activation, the nuclear factor light chain enhancer of NF-κB translocation from the cytoplasm to the nucleus, and ROS levels in HUVECs treated with ox-LDL. Interestingly, 20 nM irisin markedly improved the viability of HUVECs [23]. Through the MAPK signaling route, FNDC5/irisin promotes EC proliferation [34].

Numerous in vivo and in vitro studies demonstrated that the activation of Adenosine monophosphate-activated protein kinase (AMPK) is implicated in the anti-inflammatory response and inhibits NF-κB signaling [35]. Inhibiting NF-κB signaling and preventing the inflammatory response in arterial wall EC are two effects of the AMPK activator, metformin [36]. Irisin triggers the AMPK signaling cascade in mouse islet β-cells exposed to glucolipotoxicity and inhibits NF-κB p65 phosphorylation and inflammatory gene expression, which reduces β-cell apoptosis and restores β-cell function. Thus, in patients with obesity and type 2 diabetes, irisin may safeguard lipid metabolism and glucose homeostasis, and serve as a vascular protector.

**Figure 4.**
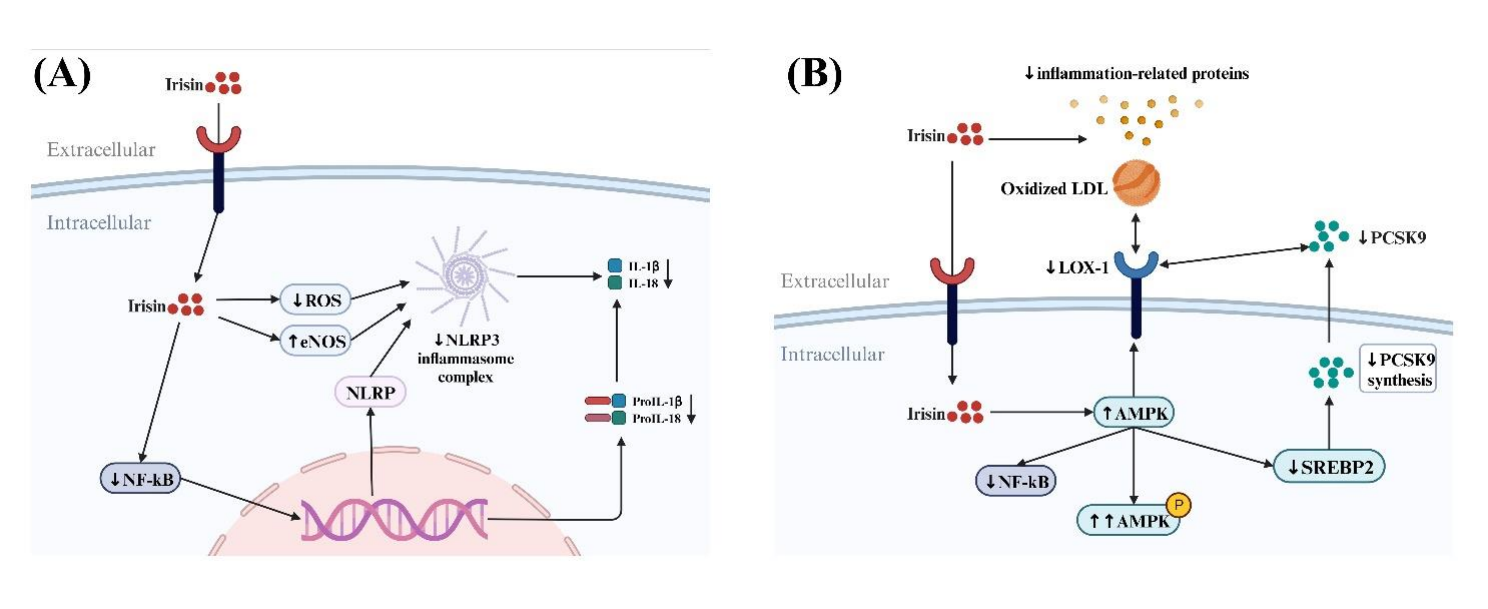
**Anti-inflammatory effect of irisin on blood vessels-NLRP3 and PCSK9. (A)** Irisin inhibits NLRP3 inflammasome activation and downregulates inflammatory factor expression. **(B)** Irisin inhibits PCSK9 expression indirectly.

### NLRP3

Irisin decreases ROS-NLRP3 inflammasome signaling, which in turn reduces inflammation and endothelial dysfunction [37, 38]. Deng et al. grew HUVECs in advanced glycation end product (AGEs) medium to examine the function of irisin and NLRP3 in AS. Based on their findings, irisin reversed the AGE-induced suppression of eNOS expression in HUVECs in a dose-dependent manner, downregulated IL -1β and interleukin-18 production, and alleviated EC and endothelial progenitor cell senescence by inhibiting ROS-NLRP3 inflammatory vesicle signaling and improving EC, endothelial progenitor cell, and VSMC function [38]. In mice with chronic kidney disease, irisin prevents vascular calcification by activating autophagy and inhibiting NLRP3-mediated pyroptosis [37] (Fig. 4A). In contrast, NLRP3 overexpression can impair the vascular protective effects of irisin [37, 38]. Irisin also prevents the activation of NLRP3 inflammatory vesicles in cardiac microvascular endothelial cells (CMECs) and treats myocardial ischemia/reperfusion injury [39].

### PCSK9

Pro-protein convertase subtilisin/kexin type 9 (PCSK9) plays a crucial role in regulating cholesterol levels. PCSK9 is primarily known for its role in regulating the metabolism of low-density lipoprotein cholesterol (LDL-C), often referred to as "bad cholesterol.” PCSK9 was found to possess pro-inflammatory properties and recent studies revealed that endothelial cells release modest levels of PCSK9 [40]. PCSK9 acts directly on the blood vessel wall, promoting an inflammatory response and exacerbating endothelial cell damage. Studies on endothelial cells revealed that PCSK9 and lectin-like ox-LDL receptor-1 (LOX-1) collaborate to cause inflammation [41]. Ox-LDL activates inflammatory pathways by upregulating LOX-1 expression in endothelial cells [42]. Irisin can protect vascular endothelial cells against the effects of ox-LDL [23, 43]. Thus, irisin may be a physiological PCSK9 inhibitor (Fig. 4B). Irisin reduced ox-LDL-induced endothelial cell inflammation by inhibiting PCSK9 expression via the AMPK/sterolregulatory element binding protein 2 (SREBP2) pathway [44].

## Anti-oxidative stress

Oxidative stress is also associated with arterial aging [14]. Impaired vascular endothelial function is directly caused by an imbalance between the generation of reactive oxygen species (ROS) and the bioavailability of NO [45]. Both human and animal studies have confirmed that oxidative stress mediates age-related endothelial dysfunction and arterial stiffness [46-49]. In the elderly population, excessive concentrations of ROS contribute to the inactivation and clearance of NO produced by ECs, thereby impairing the control of vascular tone by ECs and reducing tissue perfusion [50, 51]. Age-related atherosclerosis is exacerbated by reduced nitrogen oxide levels, which cause inflammation and leukocyte adherence [14]. ONOO^—^ in aging ECs play a role in vascular aging by impairing mitochondrial function, directly damaging cells, and in particular, activating redox-sensitive pro-inflammatory cell pathways (such as NF-κB) [52, 53]. Interestingly, ROS prevent eNOS phosphorylation, which prevents NO production, causing ECs to undergo death and promoting ONOO^—^, which damages the endothelium and accelerates AS [24, 54, 55]. By increasing vascular oxidative stress, matrix metalloproteinase (MMPs) activation and disturbance of the structural integrity of aging arteries increase aortic stiffness and promote atherogenesis [56-58].

Irisin can reduce oxidative stress in blood arteries by boosting the activity of antioxidant enzymes, such as catalase and superoxide dismutase (SOD), thereby directly promoting EC proliferation, neutralizing ROS, reducing oxidative damage to blood vessels, and safeguarding function [59]. A study on endothelial cells from type 2 diabetes mellitus (T2DM) mice found that exogenous irisin reduced endothelial protection by inhibiting the activation of the protein kinase C-β (PKC-β)/NADPH oxidase and NF-κB/Inducible nitric oxide synthase (iNOS) pathway, and the reduced levels of ONOO^—^ to decrease oxidative/nitrative stress, thereby achieving endothelial protection [60]. Similarly, elevated levels of endogenous irisin can decrease arterial stiffness in both humans and animals by increasing AMPK, Akt, and eNOS arterial phosphorylation levels, and circulating levels of nitrite/nitrate (Nox) [61]. Irisin decreased ROS levels in CMECs and attenuated H/R injury under high glucose/high fat (HG/HF) conditions [39]. Irisin boosted the amount of NO secreted by endothelial cells, which aided in the phosphorylation of eNOS, and prevented the formation of ONOO [39, 60, 62, 63]. Activation of eNOS by irisin was also concentration- and time-dependent [24]. The antioxidant effect of irisin on the endothelium might be significantly affected by the activation of the AMPK/PI3K/Akt/eNOS signaling pathway, and AMPKα has been proposed as a potential longevity-related molecule with anti-inflammatory and antioxidant properties [24, 35, 63]. Exogenous irisin treatment in spontaneously hypertensive rodents stimulated the AMPK/Akt/eNOS signaling pathway, increasing NO release and lowering blood pressure [64]. Irisin was also found to enhance endothelial function via the AMPK/Akt/eNOS signaling pathway in related studies with obese mice [65]. By increasing endogenous irisin levels, aerobic exercise markedly reduced myocardial infarction-induced inflammation in myocardial infarction-stricken mice; however, the anti-inflammatory effects of aerobic exercise were mitigated by Fndc5 deletion. Moreover, by triggering the PI3K/Akt signaling pathway, exogenous recombinant irisin significantly suppresses the inflammatory response triggered by lipopolysaccharide (LPS) [66]. Irisin reduces apoptosis during ischemia-reperfusion injury by inducing oxidative stress through the activation of the PI3K/Akt/mTOR signaling pathway [67].

### Nrf2

Nuclear factor E2 related factor 2 (Nrf2) is a key transcription factor in the endogenous antioxidant system that maintains redox homeostasis by inducing the expression of various genes involved in antioxidant defense, such as NADPH, glutathione peroxidase (GPx), NQO1, heme oxygenase-1 (HO-1) and ferritin, to maintain redox homeostasis [68]. Stimulation of Nrf2 can have positive effects on the endothelium and powerful anti-inflammatory and pro-angiogenic effects on the developing vascular system [69-71]. Compelling evidence supports the theory that Nrf2 dysfunction in the aging vascular system worsens oxidative stress and reduces the ability of vascular cells to withstand ROS-mediated cytotoxicity. A decline in vascular resistance to oxidative stress is believed to be one of the primary mediators of vascular aging [72].

Irisin shields heart microvasculature from oxidative stress and apoptosis induced by high-glucose and high-fat diets. Irisin supports CMEC proliferation and migration by stimulating Nrf2 nuclear translocation and extracellular-regulated protein kinase (ERK) phosphorylation in CMECs [73, 74]. Irisin also increases the expression of three antioxidants: SOD-1, SOD-2, and HO-1. The ERK1/2/Nrf2/antioxidant protein pathway, particularly the ERK1/2/Nrf2/HO-1 pathway, mediates the anti-apoptotic and antioxidant actions of irisin in CMECs. Irisin-induced antioxidant protein expression was inhibited by ERK1/2 inhibition or Nrf2 knockdown, eliminating the anti-apoptotic and antioxidant effects of irisin.

Angiogenesis is a crucial stage in tissue repair [75]. Therefore, irisin, a substance that can repair damaged endothelium and promote the growth of new blood vessels, may play a significant role in vascular injury caused by various factors [76]. In an in vitro study of ox-LDL-treated human umbilical vein endothelial cells, irisin increased EC viability and decreased apoptosis, counteracting the inhibitory effect of ox-LDL on angiogenesis and enabling its further promotion.

**Figure 5.**
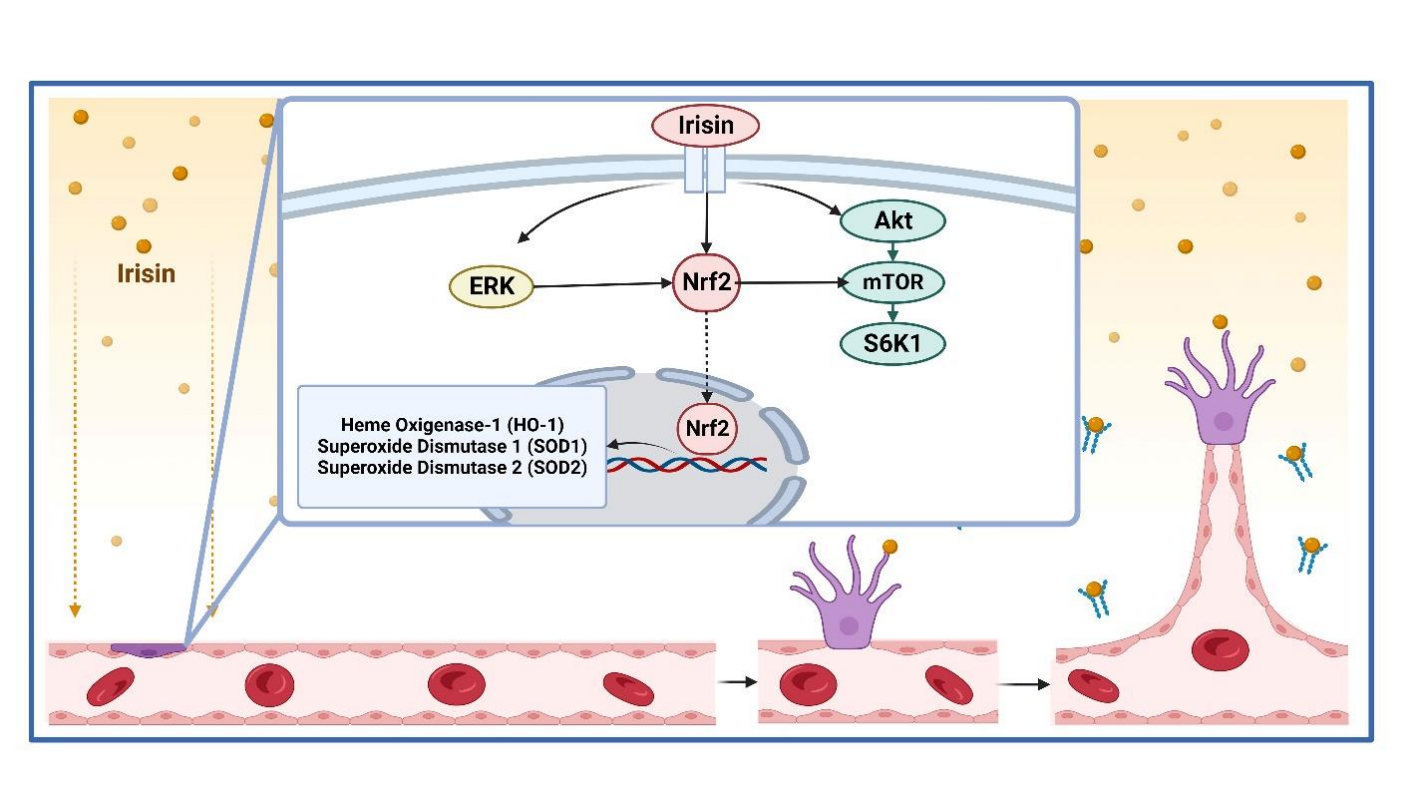
**Irisin stimulates Nrf2 to protect endothelial cells from oxidative stress and stimulates angiogenesis**. Created with BioRender.com.

The Akt/mammalian target of rapamycin (mTOR)/S6K1/Nrf2 pathway is crucial [43]. Independent of the damage sustained by the endothelium, irisin lowers the levels of inflammatory factors and ROS in ECs [43]. According to studies on the carotid arteries of rats, AMPK induces HO-1 expression in ECs via the Nrf2 signaling pathway (Fig. 5). In addition, exogenous administration of the AMPK signaling molecule, 5-aminoimidazole-4-carboxamide riboside (AICAR), increases Nrf2 expression in an AMPK-dependent manner [77].

### Macrophages

By lowering the expression of pro-inflammatory cytokines, regulating macrophage differentiation [78, 79] and chemokines in macrophages, and efficiently activating vasoprotective mechanisms that neutralize ROS, irisin has a direct impact on macrophages, a type of innate immune cell [80]. Macrophages play a key role in the generation of ROS, promotion of oxidative stress and microinflammatory states, and emergence of chronic vascular injury diseases, such as AS [62]. Irisin was found to reduce excessive ROS generation and boost macrophage proliferation in mouse macrophages cultured in an irisin-rich medium. This effect may enable normal immune functions to be performed more effectively [81].

Irisin promotes antioxidant processes mediated by Nrf2 and anti-inflammatory systems mediated by peroxisome proliferator-activated receptor gamma (PPARγ). The Nrf2 signaling pathway is an important target for AS prevention and treatment [82]. In the absence of apolipoprotein E (Apo E), Nrf2 increases AS by increasing ox-LDL uptake and foam cell formation in macrophages, and interleukin-1-mediated vascular inflammation [83]. In contrast, Nrf2-mediated antioxidant gene production may slow the onset of AS [84]. In lipopolysaccharide (LPS)-stimulated mouse macrophages, exogenous irisin treatment activated the Nrf2/HO-1 signaling axis and significantly reduced the expression and release of high-mobility group box 1 (HMGB1), effectively reducing free radical production by macrophages and exerting anti-inflammatory effects [85]. PPARγ is a target for the downregulation of pro-inflammatory cytokines via the obstruction of NF-κ B expression [86]. Exogenous FNDC5 prevents M1-type macrophage differentiation stimulated by FNDC5 deficiency [78]. Irisin possesses anti-inflammatory properties because it mechanistically inhibits the MAPK pathway and markedly increases the expression of PPARγ [87]. Furthermore, irisin was found to induce M2 macrophage differentiation through the Janus kinase 2 (JAK2)/transduction and activator of transcription 6 (STAT6)-dependent transcriptional activation of Nrf2-associated antioxidant genes and the PPARγ-related anti-inflammatory system; irisin-induced M2 macrophage differentiation was eliminated by downregulating Nrf2 and PPARγ, as confirmed in a preclinical study [79].

### Ang II

Older or angiotensin II (Ang II)-exposed mice have lower blood levels of FNDC5/irisin. Compared with wild-type mice of comparable age, arterial stiffness and senescence were more pronounced in naturally aging mice with FNDC5 deficiency [28]. However, recombinant irisin reduced the arterial stiffness and senescence induced by Ang II in vascular smooth muscle cells (VSMCs) [25]. Both in vivo and in vitro investigations have shown that irisin is a promising inhibitor of Angiotensin II (Ang II)-induced cardiotoxicity [28, 88-90]. Ang II, a key RAAS effector, lowers the antioxidant capacity of ECs, promotes apoptosis and oxidative stress by inhibiting the Nrf2/ERK1/2/NADPH oxidase 2 (Nox2) system, and harms HUVECs [69]. Based on growing evidence, RAAS upregulation induces a vascular aging phenotype, persistent inflammation, oxidative stress in the vasculature, intimal thickening, remodeling of large arteries, and increases the susceptibility of aging vessels to AS [14, 91, 92]. Irisin inhibits AngII-induced myocardial fibrosis, hypertrophy, and oxidative stress in mouse myocardium via Nrf2-mediated antioxidative stress [88]. However, the effect of irisin on Ang II-stimulated vessels has not been demonstrated, and further studies are required to understand the irisin/Nrf2/AngII mechanism in endothelial cells.

### Mitochondria

**Figure 6.**
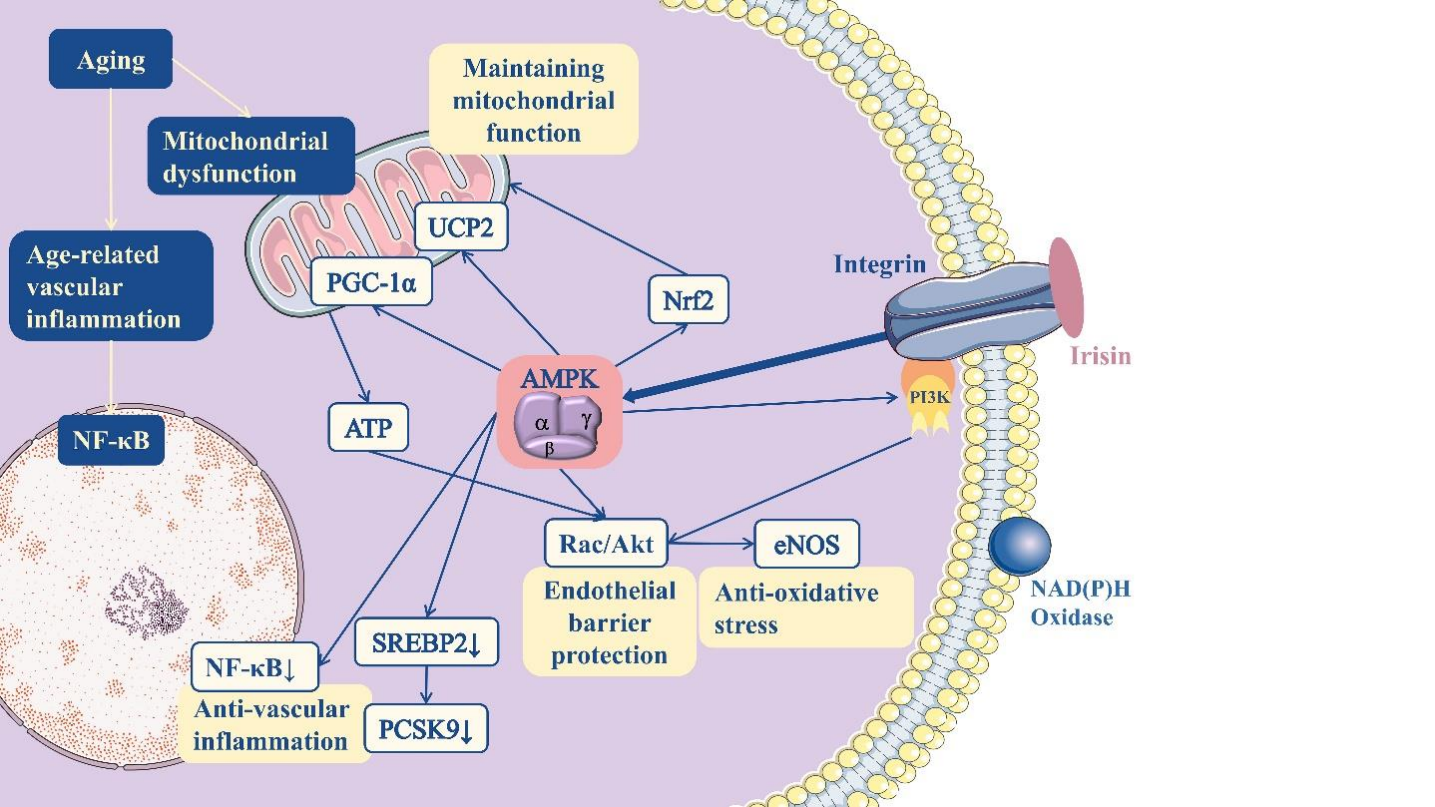
**The AMPK pathway, a highly evolutionarily conserved cellular energy state sensor that conveys metabolic stress signals and restores metabolic homeostasis by integrating physiological data, plays a role in the regulation of the vascular system by irisin**. Abbreviations: Rac/Akt- protein kinase B; NF-κB- nuclear factor-kappaB; PCSK9- pro-protein convertase subtilisin/kexin type 9; SREBP2- sterolregulatory element binding protein 2.

Maintaining sufficient mitochondrial mass and quantity in EC is a basic requirement for endothelial barrier integrity. Mitochondrial biosynthesis maintains the integrity of the vasculature, including the regulation of membrane transport and barrier function [93]. Additionally, ATP promotes Rac activation and cortical actin formation to protect the endothelial barrier, and ATP and its degradation products play significant roles in the vascular system as signaling molecules [94]. Therefore, altered mitochondrial energy metabolism or dysfunction is a key molecular and biological cause of vascular aging. ECs and VSMCs in conduit arteries and capillaries progressively develop impaired mitochondrial biosynthesis and impaired mitochondrial autophagy with age, resulting in the accumulation of dysfunctional mitochondria in vascular cells and exacerbating the vascular aging process [14]. Numerous investigations have demonstrated that PGC-1α and its downstream target, mitochondrial transcription factor A (Tfam) [1], regulate mitochondrial biogenesis, and AMPK controls PGC-1α expression [95] (Fig. 6).

### Maintenance of mitochondrial homeostasis

By reducing excessive mitochondrial fission and promoting mitochondrial fusion, irisin enhances mitochondrial dynamics in vivo and in vitro, and reduces apoptosis [67]. In mice with cerebral ischemia/reperfusion injury, irisin was found to preserve mitochondrial homeostasis by blocking mitochondrial fission-associated protein 1 (Drp1). Furthermore, mice administered irisin had a more intact mitochondrial structure [96]. Irisin also restored the expression of Optic Atrophy 1 (Opa1) and mitochondrial fusion-associated protein (Mfn2) [67]. According to a recent study, irisin prevents excessive mitochondrial fission, mediating the cardioprotective effects of exercise on the myocardium. Downregulation of endogenous irisin levels reduced exercise-induced mitochondrial fission protein expression and increased AMPK phosphorylation [97].

### Protection of the mitochondrial function

Irisin slows the development of AS and reduces early perivascular fibrosis by preventing endothelial-to-mesenchymal transition (EndMT) induced by high-glucose or cardiotoxic drugs and combating EC inflammation and oxidative stress [98, 99]. Irisin protects the mitochondria and decreases oxidative stress by increasing the production of uncoupling protein 2 (UCP2) and preventing its degradation [98, 100]. Through increased UCP2 expression in EC, irisin prevents the buildup of ROS, activation of the NF-κB-Snail pathway, EndMT phenotype change, and DIC-mediated apoptosis of cardiomyocytes. Irisin was found to protect the mitochondria during intestinal ischemia-reperfusion by activating the αⅤβ5/AMPK/UCP-2 pathway [96]. Furthermore, during cardiac ischemia-reperfusion, exogenous irisin boosted the expression of mitochondrial ubiquitin ligase (MITOL/MARCH5), which promotes mitochondrial repair and alleviates ER stress [59].

Irisin promotes mitochondrial biogenesis and uncoupling, thereby protecting EC mitochondrial function [101]. Irisin treatment markedly increased the mitochondrial number and ATP synthesis in LPS-treated ECs. Additionally, irisin may maintain the integrity of the endothelial barrier by enhancing AMPK/PGC-1α signaling, which in turn causes Nrf and its downstream receptor, Tfam, to be expressed [102, 103], thereby enhancing mitochondrial biogenesis [101]. AMPK activation can promote the expression of UCP2, which prevents hyperglycemic ECs from producing superoxide radicals and protects blood vessels. In addition, PGC-1α controls the expression of irisin, a muscle factor that is produced during PGC-1α transcription. Consequently, irisin promotes its expression via a positive feedback mechanism [104]. Interestingly, in diabetic cardiomyopathy (DCM), exogenous irisin dose variations may have the opposite effect. Low doses of irisin inhibit EndMT to protect the heart in a high-glucose environment, whereas high doses of irisin trigger MMPs, causing cardiac fibroblasts to proliferate and migrate, and ultimately degrade the myocardial physiological collagen scaffold [99]. Further studies are required to understand the mechanisms underlying the effects of irisin on the vascular endothelium as other studies have not supported the damaging effects of high irisin doses on the heart.

## Epigenetic alterations - activation of miR126-5p

Alterations in microRNAs (miRNAs) are significant epigenetic changes during vascular aging that are mediated by a wide range of epigenetic changes that occur with advancing age [14]. MiRNAs, a small class of non-coding RNAs, regulate angiogenesis in the vascular system and inhibit AS [105, 106]. In recent years, the dysregulation of miRNA expression has been proposed to be linked to age-related abnormalities in vascular function [107]. The dysregulation of miRNA expression in aging ECs and VSMCs may hinder angiogenic activity, increase cellular oxidative stress, encourage the formation of AS plaques, and render them unstable [108, 109]. The most prevalent miRNA in ECs, microRNA126 (miR126), can prevent the apoptosis of vascular endothelial cells by targeting the PI3K/Akt and MAPK signaling pathways, ultimately preventing oxidative stress and inflammation [110, 111]. According to many in vitro investigations on vascular inflammation, increased miR126 expression decreases T lymphocyte and monocyte adhesion to ECs [112].

Irisin initiates the inflammatory cascade in AS lesions by upregulating miR126, thereby targeting the inflammatory response cascade [62]. In an in vitro study, irisin treatment increased miR126-5p signaling, markedly increased HUVEC proliferation, repaired damaged endothelium, and inhibited the migration and proliferation of VSMCs [113]. The administration of exogenous irisin to Apo E-deficient mice also upregulated the expression of miR126-5p in the endothelium, increasing EC proliferation to reduce vascular remodeling, and directly inhibiting the development of AS [38, 113].

The expression of miR126-5p is dependent on ERK signaling activation. Angiogenesis is mediated by irisin-dependent ERK pathway activation, which also upregulates miR126-5p and prevents ox-LDL -impaired angiogenesis [43, 76, 114]. Irisin also stimulates cardiac angiogenesis and reduces cardiomyocyte damage by activating mitochondrial autophagy via the ERK signaling pathway [115]. Long-term exogenous irisin treatment significantly increases IGF-1 expression, which could enhance the miRNA regulation of vital target genes for vascular health [116, 117].

## A new target for delaying vascular aging

Irisin is an important chemical that aids in aging and could be a new target for extending life [34]. The direct anti-aging effects of irisin were confirmed by the strong association observed between plasma irisin levels and relative telomere length, a genetic marker of aging, in healthy individuals [118]. Since its discovery in 2012, irisin has been demonstrated to exert varying advantageous effects on target tissues. Irisin has recently been investigated as a potential target for delaying vascular aging. The most recent studies on irisin function have been conducted using preclinical models. To date, clinical investigations of the effects of irisin have been designed to investigate the impact of physical activity on endogenous irisin levels and its advantages in unhealthy situations, many of which are age-related disorders, such as CVD.

According to a recent Japanese population-based study, higher circulating irisin levels are associated with higher cardiorespiratory fitness and lower cardiometabolic risk, regardless of age or sex. In addition, endogenous irisin levels were found to be negatively correlated with percent body fat, blood pressure, fasting blood glucose, hemoglobin A1c (HbA1c), triglyceride levels, and cardiometabolic risk scores, and positively correlated with high-density lipoprotein cholesterol levels [119]. The positive effect of irisin on the blood vessels in elderly people is a growing subject of interest. Arterial stiffness is a typical issue in aging blood arteries and is associated with hypertension and CVD. Animal and human studies have revealed that irisin lowers arterial stiffness, which may assist in maintaining healthy blood pressure and lessen the burden on the heart, which may be especially advantageous for older individuals who are more likely to develop hypertension [64, 120]. Irisin has shown promise in reducing arterial stiffness in preclinical studies, which could be particularly beneficial for elderly individuals by aiding the maintenance of more youthful and flexible blood vessels [28, 61]. The structural changes that occur in blood vessels with aging, such as the thickening of vessel walls and development of atherosclerotic plaques, can be mitigated by the influence of irisin on cellular processes. Such alleviation may help maintain a healthier vascular structure in the elderly population [7, 121, 122].

Chronic inflammation and oxidative stress play important roles in vascular aging. Irisin has anti-inflammatory and antioxidant properties that may help reduce the onset of vascular aging. Exercise-induced endogenous irisin secretion is associated with improved vascular health and endothelial function in obese patients with early atherosclerosis[25]. Irisin regulates endothelial function by reducing oxidized LDL-induced endothelial damage, suppressing the expression of inflammatory and endothelial markers, and preventing the transformation of macrophages into foam cells, all of which help prevent atherosclerosis [123]. When adenovirus overexpressing FNDC5 (Ad-FNDC5) is administered to mice on a high-fat diet, elevated FNDC5/irisin levels may delay atherosclerotic plaque formation by upregulating PPAR/HO-1 signaling [124]. Coronary angiographic data from individuals older than 35 years revealed that irisin may be related to the severity of coronary artery stenosis [123]. This correlation was observed in patients with stable coronary artery disease, where endogenous irisin levels negatively correlated with the severity of coronary artery disease, as confirmed by coronary angiography [125].

Sprague-Dawley rats were implanted with an osmotic pump loaded with irisin (5 μg/kg/day) for 42 days to evaluate the effects of exogenous irisin administration on cardiac physiology. Chronic intervention with exogenous irisin increased the expression of genes associated with cardioprotection, such as FNDC5, CPT1, IGF-1, and calreticulin, and suppressed the expression of genes associated with cardiac physiology, such as PGC1, NOX4, and Mfn1 [126].

Exercise, particularly endurance and resistance training, is the most effective intervention for naturally increasing endogenous irisin levels, delaying vascular aging, and improving heart function in older individuals. When aged Sprague-Dawley male rats were subjected to twelve weeks of aerobic exercise, a considerable increase in endogenous irisin levels a reduction in cardiovascular risk, and improvement in heart function were observed [127]. A large population study revealed a strong correlation between endogenous irisin levels and leisure time physical activity (LTPA), with LTPA lowering heart rate and blood pressure by increasing irisin levels, confirming the protective role of irisin in metabolic cardiac health [128]. Furthermore, a community-based exercise program could be an effective strategy to increase irisin levels while improving muscle strength and cardiorespiratory endurance [129]. Miyamoto-Mikami et al. discovered that eight weeks of endurance exercise significantly increased serum irisin expression in healthy middle-aged and older adults [130]. However, not all older adults are ready for strenuous physical activity, and the regulation and manipulation of irisin levels, whether through exercise, medication, or other means, are complex areas with varying results in different studies. The dosages, durations, and methods for safely and effectively increasing irisin levels in humans require further exploration[113].

Of note, studies on irisin and its effects on blood vessels, particularly in elderly individuals, are ongoing. Most existing studies have been conducted using animal models, and more clinical research involving human participants is needed to better understand the applicability of these findings in real-world situations.

## Conclusions and future perspectives

The complex process of vascular aging significantly affects mortality and morbidity in the elderly population. Vascular aging involves various mechanisms at the cellular level, including epigenetic alterations and inflammation, which offer crucial targets for vascular aging intervention. Current studies lend credence to the notion that irisin not only protects against vascular aging, but also has positive effects on metabolic diseases. Reduced vascular inflammation, resistance to oxidative stress, altered epigenetics, and protection of the mitochondria are among the crucial areas in which irisin exerts its anti-vascular aging effects.

Numerous experimental studies have assessed the beneficial effects of irisin on vascular aging. The following unresolved questions and opportunities remain for future investigations with irisin. (1) Current understanding of irisin is primarily based on animal/cell experiments; however, the receptor for irisin, which is based on basic research, has yet to be identified, and the mechanism by which FNDC5 is cleaved into irisin is unclear. Further clarification of the interactions between irisin and the specific receptor, and a clearer mechanism of the induced metabolic pathways, are required; these issues will be the focus of future research. (2) No clear investigations have sought to determine whether aging-related disorders increase human irisin levels. Individual variances, half-lives, varied sample times and tissues, and different endogenous irisin assays have resulted in widely fluctuating irisin levels. However, no accurate and reproducible methods are available for detecting plasma irisin levels. As a result, detecting irisin in vivo remains a significant challenge for researchers. (3) The mechanism by which irisin controls vasodilation may involve both endothelium-dependent and non-endothelium-dependent dilation; however, some studies have suggested that irisin only controls endothelium-dependent dilation. Therefore, further studies are needed to determine whether irisin can directly control vascular function [131, 132].

The potential protective function of irisin in anti-vascular aging is still unknown, and more studies with larger clinical sample sizes, particularly prospective studies using exogenous irisin, are needed to uncover additional unknown mechanisms and other factors by which endogenous and exogenous irisin affect vascular aging in elderly people. Although irisin levels are substantially similar between species, the initial code of humans differs from that of animals, making it impossible to determine whether the findings of preclinical investigations are applicable to humans. Furthermore, in several animal and cellular experiments, the doses of irisin that exhibited biological effects were significantly higher than the physiological values, raising concerns regarding the clinical use of exogenous irisin.

In conclusion, irisin is an important mediator of anti-vascular aging. Current studies provide a precise mechanism for the anti-vascular aging effects of irisin and suggest that irisin may be an anti-vascular aging agent. However, the current understanding of vascular aging is still far from complete, and more studies are needed to demonstrate the role of irisin, a promising agent in vascular aging.
